# Supplementary material for: Association between oral microbiome and seven types of cancers in East Asian population: a two-sample Mendelian randomization analysis
Source: Front Mol Biosci. 2023 Nov 21;10:1327893. doi: 10.3389/fmolb.2023.1327893 (PMC10702768; doi:10.3389/fmolb.2023.1327893)
Supplement: Supplementary file 1 [file DataSheet1.ZIP › Supplementary Materials/MR plots of saliva/saliva═╝/Pancreatic cancer/pheno.3530_to_pancreatic cancer_forest.pdf]

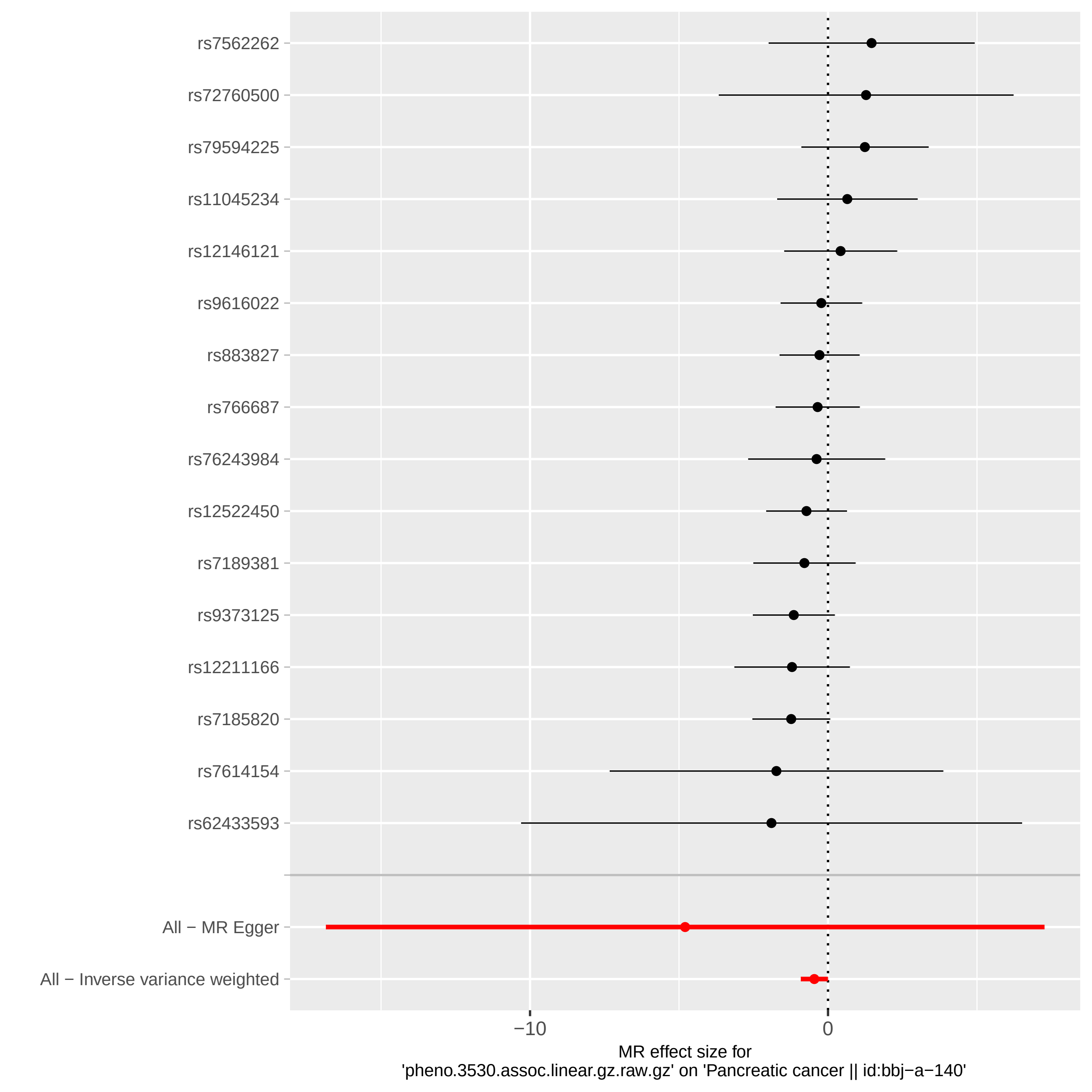
rs7562262
rs72760500
rs79594225
rs11045234
rs12146121
rs9616022
rs883827
rs766687
rs76243984
rs12522450
rs7189381
rs9373125
rs12211166
rs7185820
rs7614154
rs62433593
All − MR Egger
All − Inverse variance weighted
−10
0
MR effect size for
'pheno.3530.assoc.linear.gz.raw.gz' on 'Pancreatic cancer || id:bbj−a−140'
